# Characterizing Consumer Behavior in Leveraging Social Media for E-Patient and Health-Related Activities

**DOI:** 10.3390/ijerph16183348

**Published:** 2019-09-11

**Authors:** Ira Puspitasari, Alia Firdauzy

**Affiliations:** 1 Information System Study Program, Faculty of Science and Technology, Universitas Airlangga, Jl. Mulyorejo, Surabaya 60115, Indonesia; 2 Research Center for Quantum Engineering Design, Faculty of Science and Technology, Universitas Airlangga, Jl. Mulyorejo, Surabaya 60115, Indonesia

**Keywords:** consumer behavior, e-patient, social media for e-patient activities, social media

## Abstract

The emergence of e-patients has encouraged consumers, people who are non-medical experts, to be more engaged in healthcare needs by utilizing online sources via social media. However, the nature of social media and regulation issues have caused concerns for the reliability and validity of the shared information. These phenomena shape consumers behavior in leveraging social media for e-patient activities. This study investigates consumer behavior using an integrated model based on the Unified Theory of Acceptance and Use of Technology (UTAUT) and the Protection Motivation Theory (PMT). The data collected from the participants (N = 312) was analyzed using partial least square structural equation modelling. The results showed that behavioral intention to use social media for e-patient activities was significantly affected by performance expectancy, effort expectancy, perceived severity, perceived susceptibility, and response efficacy; and that behavioral intention corresponded positively to usage intention. In addition, the results also indicate that the intention to use social media for health-related purposes is driven by awareness of preventing health problems and attempts to reduce the risk of developing an illness. Based on findings, this study recommends strategies and initiatives to optimize social media for promoting a healthy lifestyle and educating society about public health and healthcare management.

## 1. Introduction

Advances in medical technology and greater economic pressures resulting from demographics challenges have pushed consumers, people who are non-medical professionals, to take more active roles in their own healthcare [1]. One of the most practical approaches is engaging in e-patient activities on social media [2,3,4]. Social media facilitate the creation and maintenance of supportive online health communities, provide access to consumer-friendly health education and telemedicine, reach remote areas, and allow personalization. In addition, researchers have also incorporated consumers’ social media engagement in their studies to detect and trace outbreaks [5,6,7], to improve disease surveillance systems using infodemiology data [5,6,8,9], and to improve medication management [4,10,11].

The interactive and participatory nature of social media has provided extended benefits for consumers. The use of social media for health communication and e-patient activities has been on the rise over the past few years [12,13]. In line with the rising global awareness of e-patient activities, Indonesian users, one of the largest social media users worldwide [14,15], have recently been utilizing social media for public health communication. For example, Forum Jejaring Peduli AIDS (https://www.facebook.com/Forum-Jejaring-Peduli-AIDS-110590268954574/) is an online community for HIV/AIDS care organizations and ambassadors in Indonesia. The community members use Facebook to discuss and share information and to promote empowerment for people living with HIV/AIDS. Into the Light Indonesia (www.intothelightid.org) is an online community that focuses on suicide prevention and mental health for teenagers. Forum Penderita Diabetes Indonesia (https://www.facebook.com/groups/forumpenderitadiabetesindonesia) is an online community of diabetics in Indonesia.

Despite the substantial benefits of social media for e-patient activities and health-related purposes, the reliability and validity of information shared online remains an issue [13,16,17]. Every social media user is able to create, disseminate, access, and use health information both as a content producer and/or a consumer. As content producers, users create and post health-related content based on their own/family experiences and medical history. The results vary in accuracy and quality because contents written by non-professionals often lack proper evaluation and/or rigorous review by a reputable institution. While this type of health communication might help others with similar medical conditions, it also comes with a potential risk because what works for an individual may not work for others. In addition, since anyone can be a content producer, selecting reliable and valid content on social media becomes more arduous. Health information may be misleading and this may cause serious impacts on one’s health or psyche [18,19], such as the cyberchondria phenomenon [20], i.e., the excessive fear of symptoms resulting from the review of search results and references on the Web, higher medical costs [21], and overall low quality of life due to poor health care management [22].

The use of social media for e-patient activities is increasing but consumers are becoming more and more skeptical and cautious of the reliability and validity of online health information. These phenomena shape consumers’ behavior on social media. This study aims to examine the key factors influencing consumers’ intention to adopt and use social media for e-patient activities. We developed a theoretical model based on an integration of the Unified Theory of Acceptance and Use of Technology (UTAUT) and Protection Motivation Theory (PMT) [23,24,25,26,27]. Both theories investigate consumers’ behavioral intention in adopting social media for e-patient activities. UTAUT focuses on the external variables that affect the behavior, while PMT focuses on the protective behavior in response to perceived threats or warnings. The issue of validity of health content on social media is a perceived risk that shapes the social media use for e-patient activities. As we draw the theoretical framework from UTAUT and PMT, the first research question to be addressed in this study is:

RQ1: What are the contributing factors that affect consumers’ intention in using social media for e-patient activities?

Since social media are now well established and the users are estimated to reach 302 billion worldwide in 2021 [28], more patients, families, and caregivers are turning to social media to take part in e-patient activities. This leads to the second research question:

RQ2: How can social media be optimized to facilitate e-patient activities?

## 2. Materials and Methods

Based on the Theory of Planned Behavior [29], intention captures the motivational aspects that influence the actual behavior. The stronger the intention to carry out a specific behavior, the more likely the undertaking will be. Consumers’ intention to modify or take up a specific behavior (e.g., participating in online health communities, searching and sharing health information, and having an online consultation with medical professionals) precedes and shapes the actual behavior. Accordingly, consumers’ behavior in this study refers to the intention that precedes the actual participations, which include all activities associated with e-patient and health communication on social media, and the cognitive and social processes that precede and follow these activities. The integration of UTAUT and PMT is viable to analyze and to interpret consumers’ behavior in using social media for optimum online health communication.

### 2.1. Hypotheses Development

The UTAUT provides a lens to examine users’ intentions to use information technology and their actual behavior, based on four construct variables, i.e., performance expectancy, effort expectancy, social influence, and facilitating condition [27]. The UTAUT model has been widely tested in real world applications, including healthcare technologies, to investigate the factors that influence the use and acceptance of e-health in a specific region [30,31]; consumers’ intention to adopt wearable healthcare technologies [32] and mobile health systems [33,34,35]; and healthcare professionals’ intention to adopt the mobile electronic medical record (EMR) [35]. A review in [33] suggests that perceived ease of use, perceived usefulness, and facilitating qualities influence and increase consumers’ adoption of mobile technologies. A study in [35] shows how performance expectancy affect users’ intention to use the mobile EMR and suggests that the most beneficial functions that could increase users’ performance should be optimized. A study in [34] reported that social influence was one of the significant factors that contributed to the adoption of m-health by elderly users in a developing country. The study also suggests that socio-economic and local culture might have confounded the results of their study because parts of them appeared to be inconsistent with what had been documented in the literature.

The PMT postulates that threats and coping appraisal motivate an individual to undertake self-protective behavior [25]. The PMT model has been applied to examine health-related intention and behavior, for example, the way to change attitude in embracing healthier living [36], the adoption of mobile health services [26,37], and compliance with electronic medical record privacy policies [38]. Babazadeh et al. [36] conducted a study to explore the factors that shaped skin cancer preventive behaviors (SCPB) in rural areas and suggested that it was the perceived susceptibility that influenced preventive behaviors among farmers. In another study, Guo et al. [37] reported that threat appraisal factors (perceived vulnerability and perceived severity) and coping appraisal factors (response efficacy and self-efficacy), along with the attitude specific to gender and age, affected the intention to adopt m-health. The threat appraisal factors had higher impacts on attitude among women and the elderly, while coping appraisal factors had more significant effects on attitude among men and the youth. Despite its wide application in health behavior interpretation or social media usage, the use of PMT model on social media for health communication is rather limited.

The first hypotheses set considers the consumer’s self-efficacy and the key attributes of technology acceptance, i.e., performance expectancy (PE) and effort expectancy (EE). Self-efficacy refers to the sense of confidence in one’s ability to accomplish a task successfully. The task in this study encompasses all activities of using social media for e-patient and health-related purposes. Confident and skillful users accomplish a task more frequently, thus, they perceive more benefits from using a system. Performance expectancy encompasses perceived usefulness (Technology Acceptance Model, TAM) and other constructs that are associated with the usefulness of a technology/system to assist one’s job or to improve performance. Effort expectancy is defined as the degree of ease of use of a technology/system, which includes the perceived ease of use and complexity constructs. Previous studies have demonstrated that self-efficacy positively influences PE and EE [39,40,41]. The first hypotheses set, thus, comprise:

**Hypothesis 1** **(H1).** *Self-efficacy has a positive impact on performance expectancy.*

**Hypothesis 2** **(H2).** *Self-efficacy has a positive impact on effort expectancy.*

According to the UTAUT framework, PE and EE influence the intention to use a technology, including Health Information Technology (HIT). Prior studies have demonstrated that PE and EE are positively correlated with behavioral intention, such as HIT acceptance in a specific region [30], the acceptance of wearable technology in health care [32], and the adoption of mobile technologies [34,35]. Similarly, social influence also impacts the intention to use a technology [26,42,43]. Social influence considers the social norms where an individual uses a technology/system and the influence of important others relating to the use of a technology/system [27]. Thus, the formulated hypotheses are as follows:

**Hypothesis 3** **(H3).** *Performance expectancy has a positive impact on behavioral intention to use social media for e-patient activities.*

**Hypothesis 4** **(H4).** *Effort expectancy has a positive impact on behavioral intention to use social media for e-patient activities.*

**Hypothesis 5** **(H5).** *Social influence have a positive impact on behavioral intention to use social media for e-patient activities.*

Previous studies in PMT indicate that the threat appraisal factors, i.e., perceived severity and perceived susceptibility, positively impact the intention to use a system [32,44]. Perceived susceptibility refers to how vulnerable an individual to a health threat, while perceived severity is defined as the degree of an individual’s belief that he/she would suffer from a health threat from unhealthy lifestyle [45,46]. When facing a relatively unknown or a significant threat, users tend to develop a positive attitude in taking a preventive action or in minimizing the risks. Hence, we propose the following hypotheses:

**Hypothesis 6** **(H6).** *Perceived severity has a positive impact on behavioral intention to use social media for e-patient activities.*

**Hypothesis 7** **(H7).** *Perceived susceptibility has a positive impact on behavioral intention to use social media for e-patient activities.*

Similarly, the coping factors, i.e., self-efficacy and response efficacy, also significantly influence behavioral intention [47,48]. Response efficacy refers to an individual’s belief that health threats could be reduced by adopting a specific coping response [47]. When a user decides to adopt a technology or a system, he/she considers the effort and time to learn how to use the system and how to achieve satisfactory productivity, i.e., the response cost. Therefore, a high response cost hinders technology/system adoption [44,48]. Considering the results of the PMT applications, the hypotheses are formulated as follows:

**Hypothesis 8** **(H8).** *Self-efficacy has a positive impact on behavioral intention to use social media for e-patient activities.*

**Hypothesis 9** **(H9).** *Response-efficacy has a positive impact on behavioral intention to use social media for e-patient activities.*

**Hypothesis 10** **(H10).** *Response-cost has a negative impact on behavioral intention to use social media for e-patient activities.*

Lastly, behavioral intention indicates the motivational assertion pertaining to engaging in a specific behavior that shapes the actual behavior. Drawing on the UTAUT framework, the intention to use a specific system/technology affects the actual usage [27,49], thus, we propose the following hypothesis:

**Hypothesis 11** **(H11).** *Behavioral intention has a positive impact on usage behavior.*

**Figure 1:**
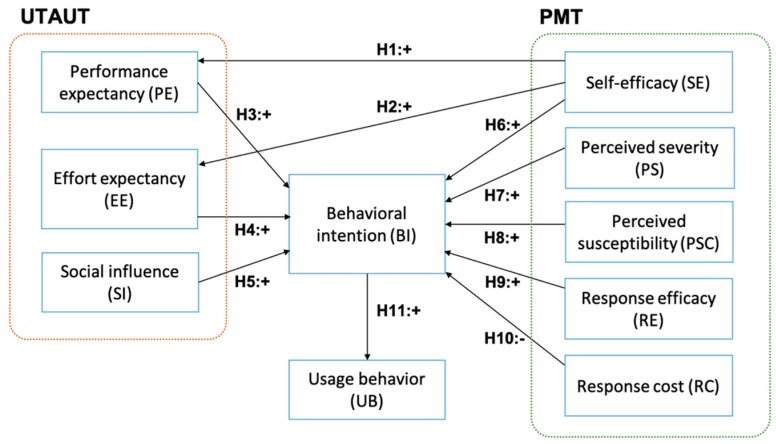
The research model to characterize the consumer behavior in leveraging social media for e-patient and health-related activities. The model was constructed based on the integrated theories of Protection Motivation Theory (PMT) and the Unified Theory of Acceptance and Use of Technology (UTAUT).

Figure 1 shows the research model of this study.

### 2.2. Data Collection

We conducted a purposive sampling to recruit the study participants from all over Indonesia. The recruited participants were non-medical professionals with a minimum age of 18 years, Indonesian citizens or have been living in Indonesia for at least one year, was on social media, at least on one platform, e.g., Twitter, Facebook, Instagram, YouTube, Blogger, etc., and had the experience in using social media for e-patient activities. The participants consisted of undergraduate and graduate students, professionals (a person engaged or qualified in a profession, i.e., lawyer, academic professor, language tutor, software engineer, and psychologist), entrepreneurs, and homemakers.

The data was collected using paper-based and web-based questionnaires that consisted of demographic profile and behavioral questions. Both questionnaires were identical. The paper-based questionnaire was given to the participants who lived in Surabaya, Jakarta, and Bandung (Indonesia), while the web-based questionnaire was sent to participants who lived outside these cities. The purpose of using the two media was to increase the distribution of the participants’ domiciles so that they adequately represented the geographical areas in Indonesia. Each questionnaire was written in two languages: English and Bahasa Indonesia. A certified translator translated the questions and all participants’ responses from Bahasa Indonesia into English. The questions relating to UTAUT were adapted from [27], while the questions pertaining to PMT were adapted from [23,50,51].

Before the data collection, one public health practitioner (Expert 1) and one health informatics expert (Expert 2) assessed the face validity (i.e., readability, feasibility, and clarity of wording) and the content validity of the questionnaire. The evaluation of the questionnaire validity followed the average congruency percentage (ACP) method [52]. Expert 1 assessed that 28 out of 29 questions were relevant, while Expert 2 agreed on 27 out of 29 questions, yielding an ACP score of 94.83%. Since the score was higher than 90%, the questionnaire was considered valid [52]. We revised the questionnaire according to the experts’ feedback on the clarity of wording and conducted a pilot readability test to 20 selected participants. Subsequently, we submitted the data collection protocol to Health Research Ethical Committee of Faculty of Public Health, Universitas Airlangga. After the ethical review approval (certificate number 50/EA/KEPK/2019), the questionnaire was distributed to the participants. The participants for paper-based questionnaire were recruited by phone, email, and via university bulletin board, while participants for the web-based questionnaire were recruited by email, messaging apps (i.e., WhatsApp and Line), and via invitation sent to Indonesian health community social media. The indicator and the corresponding question are presented in Appendix A and the full English questionnaire is provided in Supplementary Material File S1.

### 2.3. Data Model and Analysis

The data collected from the participants was tabulated using descriptive statistical analysis that covered all the demographic profiles and answers. To describe the users’ behavior in using social media for online health communication, we developed a model based on partial least square structural equation modeling (PLS-SEM). The PLS-SEM was selected because the proposed model comprised many indicators, the data collected was non-normally distributed, and one of the study purposes was to identify the driver constructs [53,54]. The evaluation of the proposed model consisted of the measurement model assessment and the structural model analysis.

The measurement model examines the indicator loadings, the internal consistency reliability, the convergent validity, and the discriminant validity [54]. The recommended indicator loadings value is above 0.708. The internal consistency can be assessed using the composite reliability (CR) and the threshold CR value is above 0.7 [55]. The convergent validity tests whether the expected related constructs shared a high proportion of the variance in the average variance extracted (AVE) metric. The recommended AVE value is higher than 0.6 [54]. Lastly, the discriminant validity tests the degree of a single construct and whether it is distinct from other constructs in the model. The discriminant validity of the construct is satisfied if the correlation between latent constructs was greater than $\sqrt[{2}]{AVE}$ [55].

Provided that all measurement model results were satisfactory, the next step was evaluating the structural model. The structural model assessment criteria included the coefficient of determination (*R*^2^), cross-validated redundancy (Q^2^), and path coefficients [54]. The *R*^2^ measures the model explanatory power within the range of 0–1, with the higher value denoting a higher explanatory power [56]. The Q^2^ assesses the model’s predictive accuracy by employing a blindfolding technique. As a guideline, the Q^2^ value should be larger than zero, with values higher than 0, 0.25, and 0.5 exhibiting small, medium, and large PLS model predictive relevance [54]. Other criteria that can be used to evaluate the structural model are the standardized root mean square residual (SRMR) and normed fit index (NFI) [57]. SRMR can be used to avoid model misspecification by measuring the difference between the observed correlation and the model implied correlation matrix [57]. The SRMR recommended value is below 0.1 or of 0.08 [58]. NFI compares the discrepancy between the χ^2^ value of the proposed model and the χ^2^ value of the null model. The general cut-off for a good fit of NFI is larger than 0.90. Finally, the last step was examining the relationships among hypothesized latent constructs and observable variables based on the statistical significance and the relevance of the path coefficients.

## 3. Results

### 3.1. Participant Profile

A total of 324 responses out of 450 targeted participants were collected from across Indonesia. The response rates of paper-based and web-based questionnaires were 78.67% (118 responses out of 150 questionnaires sent) and 68.67% (206 responses out of 300 questionnaires sent), respectively. Meanwhile, the response rates of Indonesian and English language questionnaires were 71% (284 responses out of 400 Indonesian questionnaires sent) and 80% (40 responses out of 50 English questionnaires sent), respectively. Of these, 12 questionnaires have been discarded following invalid, inconsistent and incomplete responses that have been provided in them. Therefore, the final sample included 312 valid questionnaires. We used G*Power software (Cognitive and Industrial Psychology laboratory, Heinrich-Heine-Universität, Düsseldorf, Germany.) to calculate the minimum number of sample size using a priori power analysis type test given the corresponding parameters used in this study [59]. The calculation yielded 296 sample sizes, thus, we processed all the 312 valid responses. More than 60% of the participants were female (62.18%, *n* = 194), which is in line with a previous study reporting that women were more engaged in searching for health information on the Internet than men [60]. The majority of the participants were aged 37 years and younger (76.6%, *n* = 339) and resided in provinces in Java (71.5%, *n* = 223), the country’s most populous island.

The majority of the participants had been using social media for more than six years (62.18%, *n* = 194). The top four popular social media platforms used by the participants were Instagram (*n* = 236), YouTube (*n* = 167), social blogging sites (*n* = 136), and Facebook (*n* = 130). The participants used social media to engage in a health-related discussion and communication mostly on the topic of disease information (89.10%, *n* = 278), medicine (59.29%, *n* = 185), and diet (44.23%, *n* = 138). Table 1 shows the demographic profile and Table 2 presents the distribution of social media usage among the participants.

### 3.2. Measurement Model

**Figure 2:**
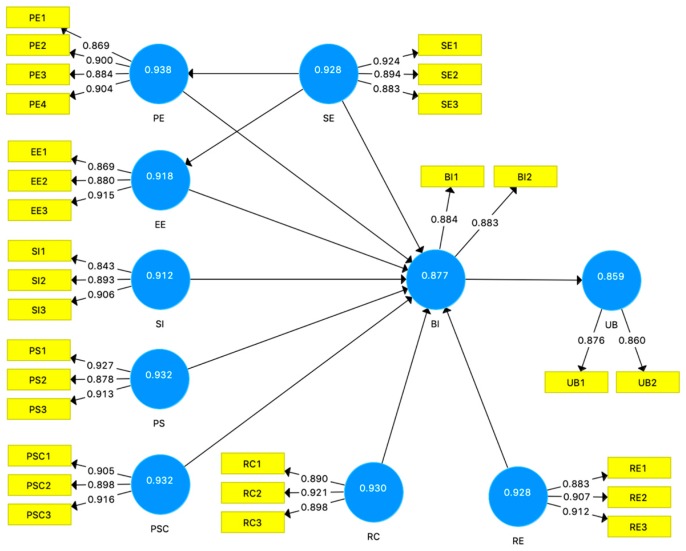
The path diagram and the measurement model. The number inside the construct shows composite reliability; and the number between construct and indicator is the loading factor of each indicator. The constructs are Performance Expectancy (PE), Effort Expectancy (EE), Self-Efficacy (SE), Social Influence (SI), Perceived Severity (PS), Perceived Susceptibility (PSC), Response Cost (RC), Response Efficacy (RE), Behavioral Intention (BI), and Usage Behavior (UB). The label inside the indicator symbol shows the indicator code for each corresponding construct.

The developed model in this study is shown in Figure 2. We applied PLS-SEM to the proposed model using SmartPLS 3.0 [61]. The criteria to assess measurement model included indicator loadings for each indicator, composite reliability and AVE for each construct, and discriminant validity. For all indicators, the loading factor ranged from 0.843 to 0.927. For all constructs, the composite reliability ranged from 0.859 to 0.938, and the AVE ranged from 0.754 to 0.821, as shown in Table 3. Hence, indicator loadings, internal consistency, and convergent validity values were higher than the recommended thresholds.

Table 4 shows the correlation value between two constructs for a discriminant validity test. All correlation values were less than the square roots of the AVE of each construct, indicating a favorable discriminant validity. Based on these results, the measurement model demonstrated satisfactory reliability and validity.

### 3.3. Structural Model

The structural model evaluation comprised the assessment of coefficient of determination (*R*^2^), the effect size *f*^2^, the cross-validated redundancy Q^2^, SRMR, and NFI. The *R*^2^ values for constructs PE, EE, BI, and UB were 0.543, 0.543, 0.842, and 0.678, respectively. The effect size of PE, EE, and UB was considered moderate, while the effect size of BI was considered substantial. The Q^2^ values for all endogenous constructs were above zero, as shown in Table 5. Constructs EE and PP exhibited medium predictive relevance, while constructs UB and BI exhibited large predictive relevance. The SRMR and NFI values were considered a good fit at 0.081 and 0.913, respectively.

### 3.4. Hypotheses Testing Results

We analyzed the model and hypothesis testing based on the structural model evaluation results, statistical significance and the relevance of the path coefficients. According to the path analysis between self-efficacy and PE (path coefficient = 0.737; *p* < 0.001), self-efficacy correlated positively with PE, which confirmed H1. A similar result between self-efficacy and EE (path coefficient = 0.737; *p* < 0.001) also suggested that high self-efficacy was correlated with high EE. This result supported H2. PE and behavioral intention exhibited a positive correlation with a path coefficient of 0.200 and *p* < 0.01, suggesting that high PE corresponded to high behavioral intention, thus confirming H3. A similar result between EE and behavioral intention (path coefficient = 0.188; *p* < 0.01) also revealed that high EE correlated with high behavioral intention, therefore supporting H4. On the contrary, there was a limited correlation between social influence and behavioral intention, as demonstrated by a path coefficient of 0.036 and *p* > 0.05. This result did not support H5.

For the next set of hypotheses, high perceived severity corresponded with high behavioral intention as exhibited by a path coefficient of 0.102 and *p* < 0.05, thus confirming H6. Similarly, perceived susceptibility correlated positively with behavioral intention (path coefficient = 0.158; *p* < 0.05), supporting H7. On the contrary, self-efficacy demonstrated a limited correlation with behavioral intention, as shown by a path coefficient of 0.044 and *p* > 0.05, thus not supporting H8.

**Figure 3:**
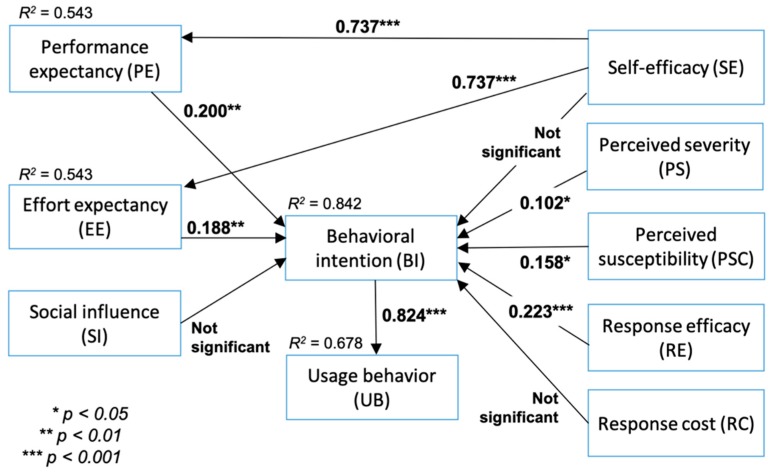
Hypothesis testing based on the structural model evaluation results, statistical significance, and the relevance of the path coefficients. Behavioral intention was significantly affected by performance expectancy (PE), effort expectancy (EE), perceived severity (PS), perceived susceptibility (PSC), and response efficacy (RE), and that behavioral intention corresponded positively to usage intention.

High response efficacy generated high behavioral intention with a path coefficient of 0.223 and *p* < 0.001, which confirmed H9. On the other hand, response cost demonstrated limited negative correlation with behavioral intention (path coefficient = −0.056; *p* > 0.05), thus not supporting H10. Finally, a high level of behavioral intention corresponded to a high level of usage intention as demonstrated by a path coefficient of 0.824 and *p* < 0.001, confirming H11. The hypothesis testing results are presented in Table 6 and summarized in Figure 3.

## 4. Discussion

The use of social media for health-related purposes is prevalent among consumers in the health sector. Despite the advantages, there is common skepticism about the reliability and the validity of health information circulating on social media. To optimize the benefits of social media for health education, this study examines how consumers leverage social media from technical and social perspectives. Results and findings from this study demonstrate that the proposed model, constructed from the integration of UTAUT and PMT, is able to characterize the consumer behavior in using social media for e-patient activities. This discussion chapter is organized into two themes: (1) consumer behavior towards adopting social media for e-patient activities to answer research question 1, (2) the implications and recommendations for leveraging social media for e-patient activities and limitations of the study to answer research question 2.

### 4.1. Consumer Behavior towards Adopting Social Media for E-Patient Activities

The positive correlation in H1 and H2 reveals that consumers who are familiar with technologies and confident with their skills tend to achieve their goals and do not require much effort to engage in online health communication and, thus, gain greater benefits. This finding is in line with other studies on online health information sharing behavior, reporting that consumers with a higher level of familiarity were likely to achieve higher search efficiency [62].

Another interesting finding is how the usefulness and the ease of use affect consumer’s behavioral intention, as shown in H3 and H4. Consumers develop their intention to continue using a system if they have a positive experience and positive impacts on their work or situation, and particularly on their health conditions [30,31,35,63]. By using social media, consumers perceived additional benefits in terms of accessing more extensive healthcare providers and health-related contents and connecting to world-wide health communities. Likewise, the ease of use of a system encourages consumers to further explore the system’s functionality similar to what has been reported by prior studies [32,34,63]. Consumers continue using and exploring social media to discover more advantages for e-patient and health-related purposes. On the cultural traits perspective, Indonesian users are willing to share beneficial information, even if it discloses some personal details, such as health conditions and medication experiences, to others. They feel appreciated and are more than happy to engage in a deeper discussion if others acknowledge the usefulness of the information they shared. Social media facilitates information sharing and the expression of appreciation among users, thus these features may encourage them to utilize the platform more frequently. On the other hand, the perception of peers’ (family members, friends) beliefs do not affect the intention to use social media for health communication, as shown by H5 rejection. This finding is in contrast to a prior study that reported social pressure influenced social media use [64]. Social media has evolved into one of the primary communication platforms for most Internet users. Therefore, the choice of social media is more likely driven by necessity than by social influence. In the case of health care, the choice of social media platform is preceded by a proper and rigorous review by the users. Users tend to be more cautious because inaccurate health information or misunderstanding health information may lead to serious health consequences. In addition, users select the social media platform based on their needs. For example, diabetic patients would join online communities related to their ailment and a consumer who suffers from a skin disease would engage in an online discussion with dermatologists in the corresponding health community.

The positive correlation in H6 reveals that consumers who are aware of the possible risks and/or threats of a health problem tend to display preventive behavior. In the case of perceived susceptibility, the awareness of health conditions and the understanding of the risks factors motivate consumers to actively broaden their knowledge of health, especially how to reduce the risks, as demonstrated by H7 acceptance. Prior studies reported that if users considered themselves more vulnerable to certain health risks/problems, then they may alter their behavior more drastically to reduce or to avoid the threat, e.g., adopting a health system device and undertaking a certain health intervention [32,36,65]. In the case of self-efficacy and behavioral intention, the H8 rejection implies that the consumer’s confidence does not affect the willingness to use social media for health communication. Consumers with high self-efficacy tend to be more aware of the risks. They tend to be skeptical of the reliability and the validity of the health information they found online [66,67]. This behavior is commonly adopted by Indonesian users because of the rising number of health-related hoaxes in Indonesian language circulating on social media [68]. Therefore, users are still cautious in using social media for health communication despite their confidence in using the system.

The result of H9 reveals that adopting specific behavior that is perceived to be effective in reducing a health problem affects the consumer’s intention to use social media. This finding is consistent with previous studies in health technology acceptance, suggesting that users would adopt health technologies when they considered that the use of the technology could reduce health threats [26,37,69]. Accordingly, based on the questionnaire results, the participants who had benefitted from social media were likely to maintain their intention to use the system. On the other hand, the rejection of H10 implies that the response cost does not affect the intention to use social media for health-related activities. This finding is in contrast with previous studies that reported a significant negative correlation between response cost and behavioral intention [26,36]. One possible explanation for the insignificant result is the consumers’ assumptions and attitudes towards the quality of health information circulating on social media. Since everyone can post health-related contents without a proper and rigorous evaluation, consumers need to double check the quality of the information on social media by reviewing the source of information, comparing the result with other sources, assessing the content accuracy, and conducting multiple clarifications with experts. The greater the effort made to crosscheck the content, the more likely consumers accept the information they found on social media. Finally, the intention to use a system greatly impacts its usage, as demonstrated in H11. The stronger the intention is, the higher the usage intensity [70].

### 4.2. Implications and Recommendations for Leveraging Social Media for E-Patient Activities and Limitations of the Study

Based on the hypothesis testing results, it could be concluded that performance expectancy, effort expectancy, perceived severity, perceived susceptibility, and response efficacy have significant impacts on behavioral intention. The strong PE and EE suggest the anticipated convenience and benefits obtained from using social media for health-related purposes. Behavioral intention is likely to increase if social media increase their ease of use and provide more advantages to the consumers. Increasing ease of use deals not only with how to use the system, but also how to interpret the health information. Social media administrators or health care organizations could provide a health terminology dictionary and organize health contents using a systematic categorization to help the consumers interpret the information.

In addition, the hypothesis testing results indicate that the intention to use social media for health-related purposes is driven by awareness of preventing a health problem and an attempt to reduce the risk of developing an illness. Consumers keep using social media, although they remain skeptical about the validity of the health information they found on social media. This finding suggests there is a great need for promotion and provision of reliable and valid health information on social media. Some possible strategies include involving medical professionals in the assessment of information quality, requiring the use of credible sources only, linking social media accounts to an accredited health institution, and requiring health content producers to be certified by a legitimate institution.

Based on the result on the coping appraisal factors, the participants believe that engaging in health-related activities on social media has helped them to achieve their goals. A high response efficacy could diminish the response cost associated with utilizing a new system. These factors could motivate consumers to keep using social media for health-related purposes. Drawing on this finding, all parties, both consumers and providers, could make greater use of social media to raise awareness about a healthy lifestyle and proactive health care management. Health care organizations and government health agencies could utilize social media to promote a healthy lifestyle, provide high quality health-related and public health contents, open online consultation and interactive discussions with medical professionals, and support e-patient communities.

While most of the results correspond to the goals of this study, future research in consumer behavioral study is required for further validation. First, applying and combining other information system theories and user behavioral models could provide valuable insight pertaining to the characterization of e-patient engagement on social media. Second, a further examination of how participants’ backgrounds affect behaviors should be addressed in future research to explore other contributing factors. Third, the participants in this study shared the same country of origin, thus, the future researches are recommended to include comprehensive studies involving participants from heterogenous background. The cultural trait attribute may affect the consumer’s behavior in communicating and disclosing health information online. Finally, future studies are recommended to continue the investigation on how the factors affecting the use of social media could improve the quality of e-patient activities and online health communication.

## 5. Conclusions

This study examines consumer behavior in leveraging social media for e-patient activities from technical and social perspectives. The significant factors that influence the usage of social media for e-patient activities are performance expectancy, effort expectancy, perceived severity, perceived susceptibility, and response efficacy. Among these factors, response efficacy and performance efficacy exert the strongest effects. It is likely for consumers to have the intention to use and to keep using a system if they reap benefits from it, such as reducing the (potential) health problems. Based on the findings, this study recommends strategies and initiatives to increase the intention to use social media for health communication, as they are one of the most accessible ways to promote a healthy lifestyle and to educate the general public about proactive health care and management.

## Figures and Tables

**Table 1:** Distribution of participants by demographic profile.

Category	N (%), N = 312
Gender	
Male	118 (37.82)
Female	194 (62.18)
Age	
18–27 years old	142 (45.51)
28–37 years old	97 (31.09)
38–47 years old	44 (14.10)
47–57 years old	21 (6.73)
58 years old and older	8 (2.57)
Occupation	
Student: high school/undergraduate/graduate	104 (33.33)
Professional	102 (32.69)
Entrepreneur	60 (19.23)
Homemaker	25 (8.01)
Other	21 (6.73)
Social Media Period of Use	
<1 year	3 (0.96)
1–3 years	23 (7.37)
4–6 years	92 (29.49)
>6 years	194 (62.18)
Geographical Distribution	
Sumatra	24 (7.7)
Java	223 (71.5)
Kalimantan	22 (7.1)
Bali and Nusa Tenggara	26 (8.3)
Sulawesi	17 (5.4)

**Table 2:** Social media platform and type of e-patient activities.

Category (N = 312)	N	Percent of Participants ^1^
Social Media Platform(s)		
Instagram	238	73.08
YouTube	167	53.53
Web blog	136	43.59
Facebook	130	41.67
Twitter	69	22.16
Other	36	11.54
E-patient Activities in Social Media		
Health information search and discussion about specific disease	278	89.10
Health information search and discussion about specific medicine	185	59.29
Health information search and discussion about diet plan	138	44.23
Access to medical professionals and health institution contact	98	31.41
Nutrition plan	77	24.68
Health information search and discussion about pregnancy	41	13.14
Access to health insurance provider	20	6.41
Other	15	4.81

^1^ the participant was permitted to select more than one answer.

**Table 3:** Loading factor, internal consistency, and convergent validity.

Variable	Indicator	Loading Factor (>0.7)	C.R. ^1^ (>0.7)	AVE ^2^ (>0.5)
Perceived Severity (PS)	PS1	0.927	0.932	0.821
	PS2	0.878		
	PS3	0.913		
Perceived Susceptibility (PSC)	PSC1	0.905	0.932	0.821
	PSC2	0.898		
	PSC3	0.916		
Response Efficacy (RE)	RE1	0.883	0.928	0.812
	RE2	0.907		
	RE3	0.912		
Self-Efficacy (SE)	SE1	0.924	0.928	0.811
	SE2	0.894		
	SE3	0.883		
Response Cost (RC)	RC1	0.890	0.930	0.816
	RC2	0.921		
	RC3	0.898		
Performance Expectancy (PE)	PE1	0.869	0.938	0.791
	PE2	0.900		
	PE3	0.884		
	PE4	0.904		
Effort Expectancy (EE)	EE1	0.869	0.918	0.789
	EE2	0.880		
	EE3	0.915		
Social Influence (SI)	SI1	0.843	0.912	0.776
	SI2	0.893		
	SI3	0.906		
Behavioral Intention (BI)	BI1	0.884	0.877	0.780
	BI2	0.883		
Usage Behavior (UB)	UB1	0.876	0.859	0.754
	UB2	0.860		

^1^ CR: Composite reliability; ^2^ AVE: Average variance extracted.

**Table 4:** Discriminant validity test: Fornell–Larcker criterion.

	BI	EE	PE	PS	PSC	RC	RE	SE	SI	UB
**BI**	0.943									
**EE**	0.830	0.927								
**PE**	0.833	0.909	0.918							
**PS**	0.833	0.741	0.738	0.938						
**PSC**	0.853	0.741	0.748	0.891	0.939					
**RC**	−0.824	−0.741	−0.742	−0.840	−0.868	0.937				
**RE**	0.851	0.731	0.740	0.858	0.903	−0.871	0.935			
**SE**	0.822	0.737	0.737	0.882	0.879	−0.847	0.854	0.935		
**SI**	0.809	0.868	0.859	0.748	0.764	−0.751	0.752	0.740	0.922	
**UB**	0.824	0.754	0.729	0.838	0.803	−0.790	0.793	0.803	0.751	0.936

**Table 5:** Predictive relevance (cross-validated redundancy).

	SSO ^1^	SSE ^2^	Q² (= 1 − SSE/SSO)
BI	624,000	182,825	0.707
EE	936,000	523,378	0.441
PE	1,248,000	713,042	0.429
UB	624,000	268,644	0.569

^1^ SSE: sum of squares of prediction errors; ^2^ SSO: sum of squares of observations.

**Table 6:** The significance of the relationships in the model.

	Original Sample (O)	Sample Mean (M)	Standard Deviation (STDEV)	T Statistics (|O/STDEV|)	*p* Values
BI -> UB	0.824	0.822	0.020	40.450	**0.000**
EE -> BI	0.188	0.188	0.064	2.937	**0.002**
PE -> BI	0.200	0.199	0.079	2.525	**0.006**
PS -> BI	0.102	0.101	0.058	1.759	**0.039**
PSC -> BI	0.158	0.154	0.074	2.136	**0.016**
RC -> BI	−0.056	−0.057	0.056	0.993	0.160
RE -> BI	0.223	0.225	0.062	3.598	**0.000**
SE -> BI	0.044	0.047	0.056	0.775	0.219
SE -> EE	0.737	0.734	0.027	26.915	**0.000**
SE -> PE	0.737	0.734	0.030	24.869	**0.000**
SI -> BI	0.036	0.035	0.056	0.642	0.261

## Supplementary Materials

The following are available online at https://www.mdpi.com/1660-4601/16/18/3348/s1, File S1. Questionnaire.

## Appendix A

**Table A1:** Variable, indicator, and the corresponding question used in the questionnaire.

Variable	Indicator	Question
Perceived Severity (PS)	PS1	I believe that if I was unresponsive to be aware of a serious disease, the prevention and the treatment would be more difficult.
	PS2	I believe that serious disease would impact my whole life.
	PS3	I would feel distressed to get a serious disease.
Perceived Susceptibility (PSC)	PSC1	My chances of getting a serious disease is high.
	PSC2	Getting a serious disease is a big concern for me.
	PSC3	I feel more vulnerable to a serious disease than others.
Response Efficacy (RE)	RE1	Using social media for e-patient and health-related activities would help me detect a serious disease early.
	RE2	Engaging in e-patient and health-related activities in social media would help me monitor my health.
	RE3	Engaging in e-patient and health-related activities in social media would help me recognize my health condition.
Self-Efficacy (SE)	SE1	I believe that I would use social media for e-patient and health-related activities
	SE2	I feel confident that I would be able to operate social media for e-patient and health-related activities.
	SE3	I feel confident with my ability to use social media, even without any guidelines on how to use it.
Response Cost (RC)	RC1	Using social media for e-patient and health-related activities requires a lot of time
	RC2	Using social media for e-patient and health-related activities would change my lifestyle.
	RC3	Using social media for e-patient and health-related activities is inconvenient.
Performance Expectancy (PE)	PE1	Using social media for e-patient and other health-related activities would help me understand health issues that matter to me.
	PE2	Using social media for e-patient and health-related activities will would me track health issues that matter to me.
	PE3	Using social media for e-patient and health-related activities would assist me obtain feedback and advice from medical professionals and other consumers.
	PE4	Overall, using social media for e-patient and health-related activities would improve my healthcare management.
Effort Expectancy (EE)	EE1	Using social media for e-patient and health-related activities would be easy for me
	EE2	I feel familiar with social media features to access health information.
	EE3	It would be easy for me to become skillful at using social media for e-patient and health-related activities.
Social Influence (SI)	SI1	My family and friends use social media for e-patient and health-related activities.
	SI2	According to my family and friends, I should use social media for e-patient and health-related activities.
	SI3	I use social media for e-patient and health-related activities because my family and my friends also use it.
Behavioral Intention (BI)	BI1	I would like to continue to use social media for e-patient and health-related activities
	BI2	My intention to use social media for e-patient and health-related activities is high.
Usage Behavior (UB)	UB1	I frequently use social media for e-patient and health-related activities.
	UB2	I explore and use many features of social media for e-patient and health-related activities.

## References

[1] Clemensen J., Danbjørg D.B., Syse M.D., Coxon I.R. The rise of patient 3.0: The impact of social media. Proceedings of the 8th International Conference on E-Health, EH 2016—Part of the Multi Conference on Computer Science and Information Systems 2016.

[2] Buchanan R., Beckett R.D. (2014). Assessment of vaccination-related information for consumers available on Facebook^®^. Health Inf. Libr. J..

[3] Grajales F.J., Sheps S., Ho K., Novak-Lauscher H., Eysenbach G. (2014). Social media: A review and tutorial of applications in medicine and health care. J. Med. Internet Res..

[4] Househ M., Borycki E., Kushniruk A. (2014). Empowering patients through social media: The benefits and challenges. Health Inform. J..

[5] Nsoesie E.O., Flor L., Hawkins J., Maharana A., Skotnes T., Marinho F., Brownstein J.S. (2016). Social media as a sentinel for disease surveillance: What does sociodemographic status have to do with it?. PLoS Curr..

[6] Santillana M., Nguyen A.T., Dredze M., Paul M.J., Nsoesie E.O., Brownstein J.S. (2015). Combining search, social media, and traditional data sources to improve influenza surveillance. PLoS Comput. Biol..

[7] Yom-Tov E., Borsa D., Cox I.J., McKendry R.A. (2014). Detecting disease outbreaks in mass gatherings using Internet data. J. Med. Internet Res..

[8] Broniatowski D.A., Paul M.J., Dredze M. (2013). National and local influenza surveillance through twitter: An analysis of the 2012–2013 influenza epidemic. PLoS ONE.

[9] Nagar R., Yuan Q., Freifeld C.C., Santillana M., Nojima A., Chunara R., Brownstein J.S. (2014). A case study of the New York City 2012–2013 influenza season with daily geocoded Twitter data from temporal and spatiotemporal perspectives. J. Med. Internet Res..

[10] Välimäki M., Athanasopoulou C., Lahti M., Adams C.E. (2016). Effectiveness of social media interventions for people with schizophrenia: A systematic review and meta-analysis. J. Med. Internet Res..

[11] Lee K., Agrawal A., Choudhary A. Mining social media streams to improve public health allergy surveillance. Proceedings of the 2015 IEEE/ACM International Conference on Advances in Social Networks Analysis and Mining 2015—ASONAM’15.

[12] Zhao Y., Zhang J. (2017). Consumer health information seeking in social media: A literature review. Health Inf. Libr. J..

[13] Song H., Omori K., Kim J., Tenzek K.E., Morey Hawkins J., Lin W.-Y., Kim Y.-C., Jung J.-Y. (2016). Trusting social media as a source of health information: Online surveys comparing the United States, Korea, and Hong Kong. J. Med. Internet Res..

[14] Allin1Social Allin1Social—Free Facebook Statistics by Country Allin1Social. https://www.statista.com/statistics/268136/top-15-countries-based-on-number-of-facebook-users/.

[15] Statista Number of Social Network Users in Selected Countries in 2018 and 2023 (in Millions). https://www.statista.com/statistics/278341/number-of-social-network-users-in-selected-countries/.

[16] Dalmer N.K. (2017). Questioning reliability assessments of health information on social media. J. Med. Libr. Assoc..

[17] Tunnecliff J., Ilic D., Morgan P., Keating J., Gaida J.E., Clearihan L., Sadasivan S., Davies D., Ganesh S., Mohanty P. (2015). The acceptability among health researchers and clinicians of social media to translate research evidence to clinical practice: Mixed-methods survey and interview study. J. Med. Internet Res..

[18] Puspitasari I. The impacts of consumer’s health topic familiarity in seeking health information online. Proceedings of the 2017 15th IEEE/ACIS International Conference on Software Engineering Research, Management and Applications, SERA 2017.

[19] Puspitasari I., Fukui K.-I., Moriyama K., Numao M. (2014). Predicting consumer familiarity with health topics by query formulation and search result interaction. Pacific RIM International Conference on Artificial Intelligence.

[20] Starcevic V., Berle D. (2013). Cyberchondria: Towards a better understanding of excessive health-related Internet use. Expert Rev. Neurother..

[21] Montgomery L. (2015). Supporting radiation therapy patients with limited health literacy. J. Med. Imag. Radiat. Sci..

[22] Mackert M., Mabry-Flynn A., Champlin S., Donovan E.E., Pounders K. (2016). Health literacy and health information technology adoption: The potential for a new digital divide. J. Med. Internet Res..

[23] Hsieh H.-L., Kuo Y.-M., Wang S.-R., Chuang B.-K., Tsai C.-H. (2016). A study of personal health record user’s behavioral model based on the PMT and UTAUT integrative perspective. Int. J. Environ. Res. Pub. Health.

[24] Maruping L.M., Bala H., Venkatesh V., Brown S.A. (2017). Going beyond intention: Integrating behavioral expectation into the unified theory of acceptance and use of technology. J. Assoc. Inf. Sci. Technol..

[25] Rogers R.W. (1975). A protection motivation theory of fear appeals and attitude change1. J. Psychol..

[26] Sun Y., Wang N., Guo X., Peng Z. (2013). Understanding the acceptance of mobile health services: A comparison and integration of alternative models. J. Electron. Commer. Res..

[27] Venkatesh V., Morris M.G., Davis G.B., Davis F.D. (2003). User acceptance of information technology: Toward a unified view. MIS Q..

[28] Wagner P. By 2021 More Than 1/3 of the Globe Will be on Social Media. https://www.statista.com/chart/15355/social-media-users/.

[29] Ajzen I. (1991). The theory of planned behavior. Organ. Behav. Hum. Decis. Process..

[30] Aldosari B. (2012). User acceptance of a Picture Archiving and Communication System (PACS) in a Saudi Arabian hospital radiology department. BMC Med. Inf. Decis. Mak..

[31] Jewer J. (2018). Patients’ intention to use online postings of ED wait times: A modified UTAUT model. Int. J. Med. Inf..

[32] Gao Y., Li H., Luo Y. (2015). An empirical study of wearable technology acceptance in healthcare. Ind. Manag. Data Syst..

[33] Garavand A., Samadbeik M., Kafashi M., Abhari S. (2017). Acceptance of health information technologies, acceptance of mobile health: A review article. J. Biomed. Phys. Eng..

[34] Hoque R., Sorwar G. (2017). Understanding factors influencing the adoption of mHealth by the elderly: An extension of the UTAUT model. Int. J. Med. Inf..

[35] Kim S., Lee K.-H., Hwang H., Yoo S. (2015). Analysis of the factors influencing healthcare professionals’ adoption of mobile electronic medical record (EMR) using the unified theory of acceptance and use of technology (UTAUT) in a tertiary hospital. BMC Med. Inf. Decis. Mak..

[36] Babazadeh T., Nadrian H., Banayejeddi M., Rezapour B. (2017). Determinants of skin cancer preventive behaviors among rural farmers in Iran: An application of protection motivation theory. J. Cancer Educ..

[37] Guo X., Han X., Zhang X., Dang Y., Chen C. (2015). Investigating m-health acceptance from a protection motivation theory perspective: Gender and age differences. Telemed. E-Health.

[38] Sher M.-L., Talley P.C., Yang C.-W., Kuo K.-M. (2017). Compliance with electronic medical records privacy policy: An empirical investigation of hospital information technology staff. Inq. J. Health Care Organ. Provis. Financ..

[39] Chiu Y.-L., Tsai C.-C. (2014). The roles of social factor and internet self-efficacy in nurses’ web-based continuing learning. Nurse Educ. Today.

[40] Liaw S.-S., Huang H.-M. (2013). Perceived satisfaction, perceived usefulness and interactive learning environments as predictors to self-regulation in e-learning environments. Comput. Educ..

[41] Kim J., Park H.-A. (2012). Development of a health information technology acceptance model using consumers’ health behavior intention. J. Med. Internet Res..

[42] Jackson J.D., Yi M.Y., Park J.S. (2013). An empirical test of three mediation models for the relationship between personal innovativeness and user acceptance of technology. Inf. Manag..

[43] Ketikidis P., Dimitrovski T., Lazuras L., Bath P.A. (2012). Acceptance of health information technology in health professionals: An application of the revised technology acceptance model. Health Inform. J..

[44] Vance A., Siponen M., Pahnila S. (2012). Motivating IS security compliance: Insights from habit and protection motivation theory. Inf. Manag..

[45] Milne S., Sheeran P., Orbell S. (2000). Prediction and intervention in health-related behavior: A meta-analytic review of protection motivation theory. J. Appl. Soc. Psychol..

[46] Maddux J.E., Rogers R.W. (1983). Protection motivation and self-efficacy: A revised theory of fear appeals and attitude change. J. Exp. Soc. Psychol..

[47] Siponen M., Adam Mahmood M., Pahnila S. (2014). Employees’ adherence to information security policies: An exploratory field study. Inf. Manag..

[48] Anderson C.L., Agarwal R. (2010). Practicing safe computing: A multimethod empirical examination of home computer user security behavioral intentions. MIS Q..

[49] Venkatesh V., Thong J.Y.L., Xu X. (2016). Unified theory of acceptance and use of technology: A synthesis and the road ahead. J. Assoc. Inf. Syst..

[50] Sun Young Lee S.Y., Hwang H., Hawkins R., Pingree S. (2008). Interplay of negative emotion and health self-efficacy on the use of health information and its outcomes. Commun. Res..

[51] Greene K., Rubin D.L., Hale J.L., Walters L.H. (1996). The utility of understanding adolescent egocentrism in designing health promotion messages. Health Commun..

[52] Popham W.J. (1978). Criterion-Referenced Measurement.

[53] Hair J.F., Risher J.J., Sarstedt M., Ringle C.M. (2019). When to use and how to report the results of PLS-SEM. Eur. Bus. Rev..

[54] Hair J.F., Hult G.T.M., Ringle C.M., Sarstedt M. (2016). A Primer on Partial Least Squares Structural Equation Modeling (PLS-SEM).

[55] Hair J.F., Black W.C., Babin B.J., Anderson R.E. (2010). Multivariate Data Analysis.

[56] Shmueli G., Koppius O.R. (2011). Predictive analytics in information systems research. MIS Q..

[57] Henseler J., Dijkstra T.K., Sarstedt M., Ringle C.M., Diamantopoulos A., Straub D.W., Ketchen D.J., Hair J.F., Hult G.T.M., Calantone R.J. (2014). Common beliefs and reality about PLS: Comments on Rönkkö and Evermann (2013). Organ. Res. Methods.

[58] Hu L.T., Bentler P.M. (1999). Cutoff criteria for fit indexes in covariance structure analysis: Conventional criteria versus new alternatives. Struct. Equ. Model..

[59] Franz F., Edgar E., Albert-Georg L., Axel B. (2007). G*Power 3: A flexible statistical power analysis program for the social, behavioral, and biomedical sciences. Behav. Res. Methods.

[60] Bidmon S., Terlutter R. (2015). Gender differences in searching for health information on the internet and the virtual patient-physician relationship in Germany: Exploratory results on how men and women differ and why. J. Med. Internet Res..

[61] Ringle C.M., Wende S., Becker J.M. SmartPLS 3. Bönningstedt: SmartPLS. http://www.smartpls.com.

[62] Puspitasari I., Moriyama K., Fukui K.-I., Numao M. (2015). Effects of individual health topic familiarity on activity patterns during health information searches. JMIR Med. Inform..

[63] Cimperman M., Makovec Brenčič M., Trkman P. (2016). Analyzing older users’ home telehealth services acceptance behavior-applying an Extended UTAUT model. Int. J. Med. Inf..

[64] Gruzd A., Staves K., Wilk A. (2012). Connected scholars: Examining the role of social media in research practices of faculty using the UTAUT model. Comput. Hum. Behav..

[65] Zhao Y., Ni Q., Zhou R. (2018). What factors influence the mobile health service adoption? A meta-analysis and the moderating role of age. Int. J. Inf. Manag..

[66] Fischer S.H., David D., Crotty B.H., Dierks M., Safran C. (2014). Acceptance and use of health information technology by community-dwelling elders. Int. J. Med. Inf..

[67] Chaudhuri S., Le T., White C., Thompson H., Demiris G. (2013). Examining health information-seeking behaviors of older adults. Comput. Inf. Nurs..

[68] Masyarakat Telematika Indonesia Hasil Survey Wabah Hoax Nasional 2019. https://mastel.id/hasil-survey-wabah-hoax-nasional-2019/.

[69] Zhang X., Han X., Dang Y., Meng F., Guo X., Lin J. (2017). User acceptance of mobile health services from users’ perspectives: The role of self-efficacy and response-efficacy in technology acceptance. Inform. Health Soc. Care.

[70] Iqbal U., Ho C.H., Li Y.C., Nguyen P.A., Jian W.S., Wen H.C. (2013). The relationship between usage intention and adoption of electronic health records at primary care clinics. Comput. Methods Programs Biomed..

