# Supplementary material for: Characterizing Consumer Behavior in Leveraging Social Media for E-Patient and Health-Related Activities
Source: Int J Environ Res Public Health. 2019 Sep 11;16(18):3348. doi: 10.3390/ijerph16183348 (PMC6765822; doi:10.3390/ijerph16183348)
Supplement: Supplementary file 1 [file ijerph-16-03348-s001.pdf]

International Journal of
Environmental Research and Public Health

Supplementary Materials: Characterizing Consumer Behavior in Leveraging Social Media for E-Patient and Health-Related Activities

Ira Puspitasari [1,2,*] and Alia Firdauzy [3]

1 Information System Study Program, Faculty of Science and Technology, Universitas Airlangga, Surabaya, Indonesia
2 Research Center for Quantum Engineering Design, Faculty of Science and Technology, Universitas Airlangga, Surabaya, Indonesia
3 Information System Study Program, Faculty of Science and Technology, Universitas Airlangga, Surabaya, Indonesia; alia.firdauzy-13@fst.unair.ac.id
* Correspondence: ira-p@fst.unair.ac.id; Tel.: +62-31-596-5257

File S1. Consumer behavior in leveraging social media for e-patient activities questionnaire.

Dear participant,

The evolution of social media and the emergence of e-patient movement have encouraged global consumers to use social media for e-patient and health-related activities. This survey aims to examine how you engage in social media for health-related purposes. Your answer will be helpful in improving the quality of online health communication and e-patient activities via social media. Your responses will be confidential and will be used for research purpose only.

Thank you very much for your participation.

Demographic Profile

Gender	Male	Female			
Age	18–27	28–37	38–47	48–57	≥58
Occupation	Student: high school, undergraduate, graduate				
	Professional (e.g., academic professor, language tutor, psychologist, software engineer etc.) Please specify:				
	Entrepreneur				
	Housewife				
	Other: …………………………….……				
Domicile city	……………………………………………				
Domicile province	……………………………………………				
Social media period of use	<1 year	1–3 years	4–6 years	>6 years	

Social Media Profile

What social media platform(s) do you use for e-patient and health-related activities? Please select all answers that apply.

Facebook	YouTube
Twitter	Web-blog
Instagram	Other: ……………………………………………

How do you use social media for e-patient and health-related activities? Please select all answers that apply.

Health information search and discussion about specific disease

Health information search and discussion about specific medicine

Health information search and discussion about diet plan

Access to medical professionals and health institution contact

Nutrition plan

Health information search and discussion about pregnancy

Access to health insurance provider

Other: ……………………………………………………………………………………………………….

Health Perception and E-patient Activities in Social Media

Please check (√) one answer of each statement which corresponds most closely to your agreement / disagreement based on the following scale:

1: strongly disagree; 2: disagree; 3: somewhat disagree; 4: somewhat agree; 5: agree; 6: strongly agree

Question	1	2	3	4	5	6
**Perceived severity**						
I believe that if I was unresponsive to be aware of a serious disease, the prevention and the treatment would be more difficult						
I believe that serious disease would impact my whole life						
I would feel distressed to get a serious disease						
**Perceived susceptibility**						
My chances of getting a serious disease is high						
Getting a serious disease is a big concern for me						
I feel more vulnerable to a serious disease than others						
**Response efficacy**						
Using social media for e-patient and health-related activities would help me detect a serious disease early						
Engaging in e-patient and health-related activities in social media would help me monitor my health						
Engaging in e-patient and health-related activities in social media would help me recognize my health condition						
**Self-efficacy**						
I believe that I would use social media for e-patient and health-related activities						
I feel confident that I would be able to operate social media for e-patient and health-related activities						
I feel confident with my ability to use social media, even without any guidelines on how to use it						
**Response cost**						
Using social media for e-patient and health-related activities requires a lot of time						

Question	1	2	3	4	5	6
Using social media for e-patient and health-related activities would change my lifestyle						
Using social media for e-patient and health-related activities is inconvenient						
**Performance expectancy**						
Using social media for e-patient and other health-related activities would help me understand health issues that matter to me						
Using social media for e-patient and health-related activities will would me track health issues that matter to me						
Using social media for e-patient and health-related activities would assist me obtain feedback and advice from medical professionals and other consumers						
Overall, using social media for e-patient and health-related activities would improve my healthcare management.						
**Effort expectancy**						
Using social media for e-patient and health-related activities would be easy for me						
I feel familiar with social media features to access health information						
It would be easy for me to become skillful at using social media for e-patient and health-related activities						
**Social influence**						
My family and friends use social media for e-patient and health-related activities						
According to my family and friends, I should use social media for e-patient and health-related activities						
I use social media for e-patient and health-related activities because my family and my friends also use it						
**Behavioral intention**						
I would like to continue to use social media for e-patient and health-related activities						
My intention to use social media for e-patient and health-related activities is high						
**Usage behavior**						
I frequently use social media for e-patient and health-related activities						
I explore and use many features of social media for e-patient and health-related activities						
